# Retrospective Multi-Locus Sequence Analysis of African Swine Fever Viruses by “PACT” Confirms Co-Circulation of Multiple Outbreak Strains in Uganda

**DOI:** 10.3390/ani14010071

**Published:** 2023-12-24

**Authors:** Tonny Kabuuka, Henry Mulindwa, Armanda D. S. Bastos, Juanita van Heerden, Livio Heath, Folorunso O. Fasina

**Affiliations:** 1 Infectious Animal Diseases Laboratory, National Livestock Resources Research Institute (NaLIRRI), National Agricultural Research Organisation (NARO), Totoro 21403, Uganda; henry.mulindwa@naro.go.ug; 2 Department of Production Animal Studies (DPAS), Faculty of Veterinary Science, University of Pretoria, Onderstepoort 0110, South Africa; 3 Department of Zoology and Entomology, Faculty of Natural and Agricultural Sciences, University of Pretoria, Hatfield 0028, South Africa; armanda.bastos@up.ac.za; 4 Department of Veterinary Tropical Diseases, Faculty of Veterinary Science, University of Pretoria, Onderstepoort 0110, South Africa; 5 Transboundary Animal Diseases Programme, Agricultural Research Centre-Onderstepoort Veterinary Institute, Onderstepoort 0110, South Africa; vanheerdenj@arc.agric.za (J.v.H.); heathl@arc.agric.za (L.H.); 6 Food and Agriculture Organization of the United Nations (FAO), I-00100 Rome, Italy

**Keywords:** conventional PCR, ELISA, diagnosis, *p54* gene, *p72* gene, central variable region (CVR), *9RL* ORF, *TK* gene

## Abstract

**Simple Summary:**

African swine fever (ASF) is a disease that affects and kills pigs in large numbers causing untold hardship to pig farmers. To limit the impact of this disease, it is important to collect samples from sick pigs, and conduct confirmatory laboratory testing to ensure that disease control measures are implemented rapidly to limit spread. In this study, we compare six laboratory tests (one serological and five conventional PCR assays) in pigs. During active infections, PCR screening of DNA extracted from tissue samples obtained from abattoirs outperformed ELISA detection of antibodies in serum samples from live pigs. Of the five PCR assays assessed, the ASF virus detection capabilities of the thymidine kinase (TK) gene assay outperformed all other conventional PCR assays assessed.

**Abstract:**

African swine fever (ASF) is a haemorrhagic fever of swine that severely constrains pig production, globally. In Uganda, at least 388 outbreaks of ASF were documented from 2001 to 2012. We undertook a retrospective serological and molecular survey of ASF virus (ASFV) using banked samples collected from seven districts (Pallisa, Lira, Abim, Nebbi, Kabarole, Kibaale, and Mukono) of Uganda. Six assays (ELISA for antibody detection, diagnostic *p72* gene PCR and genomic amplification, and sequencing of four gene regions (*p72* [P], *p54* [A], CVR of the *9RL*-ORF [C], and *TK* [T]), hereinafter referred to as P-A-C-T (PACT)) were evaluated. Antibodies to ASFV were detected in the Abim district (6/25; 24.0%), and the remainder of the serum samples were negative (187/193; 96.9%). For the tissue samples, ASFV detection by assay was 8.47% for P, 6.78% for A, 8.47% for C, and 16.95% for T. The diagnostic PCR (*p72* gene) detected seven positive animals from four districts, whereas the *TK* assay detected ten positives from all seven districts. In addition to the superior detection capability of TK, two virus variants were discernible, whereas CVR recovered three variants, and *p72* and *p54* sequencing each identified a single variant belonging to genotype IX. Our results indicate that dependence on serology alone underestimates ASF positivity in any infected region, that multi-locus sequence analysis provides better estimates of outbreak strain diversity, and that the *TK* assay is superior to the WOAH-prescribed conventional *p72* diagnostic PCR, and warrants further investigation.

## 1. Introduction

African swine fever (ASF) is an important transboundary animal disease affecting swine, and has morbidity and mortality rates approaching 100%. In the continued absence of an effective vaccine and treatment options, it remains a devastating threat to the pig industry [1,2]. The disease is caused by the ASF virus (ASFV), an arthropod-borne virus belonging to the family *Asfaviridae* and genus *Asfivirus*. Since its initial identification in pigs in Kenya in 1921 [3], it has been reported in Uganda, where it is now endemic. ASF is caused by a complex and large enveloped DNA virus with a genome of 170–190 kbp [4]. Up to 24 different genotypes have been shown to occur in sub-Saharan Africa based on C-terminal *p72* sequencing [5,6,7,8].

A high degree of genetic variability of the virus in its endemic setting has been shown to occur [5,6,7,8]. The virus is harboured naturally in both vertebrate and invertebrate sylvatic hosts throughout sub-Saharan Africa, where it is transmitted to domestic pigs when infected soft-shelled, eyeless ticks of the *Ornithodoros moubata* complex feed on them [9,10]. ASFV can survive for more than five years in colonies of competent soft tick arthropod vectors of the *Ornithodoros* genus that have not been exposed to an infectious bloodmeal, and act as a source of infection for wild and domestic pigs [11,12]. While endemic African suids such as warthogs (*Phacocoerus africanus* and *Ph. aethiopicus*), bush pigs (*Potamochoerus larvatus*), the Red river hog (*Po. porcus*), and giant forest hogs (*Hylochoerus meinertzhangeni*) can be infected, they do not exhibit clinical symptoms [13]. In contrast, European wild boar (*Sus scrofa*) and feral pigs as well as domestic pigs are equally susceptible to ASFV and show similar clinical signs and mortality patterns [14].

In East Africa, there is a sylvatic cycle involving warthogs and *Ornithodoros* soft ticks, as well as maintenance of the virus in the absence of wild pigs and soft ticks. Moreover, Haresnape & Wilkinson [15] showed the maintenance of ASF virus in a cycle between domestic pigs and *Ornithodoros* inhabiting the pigsties in Malawi. The presence of all three epidemiological cycles in the region complicates control of the disease.

Over a twelve-year period (2001–2012) in Uganda, at least 388 outbreaks of ASF were documented [16,17]. Seven haemadsorbing viruses were isolated, and all were classified within the domestic pig cycle-associated *p72* and *p54* genotype IX [18]. Another study in Uganda indicated that domestic pigs, bushpigs, warthogs, and soft ticks may have played various roles in the epidemiology of ASF, with some pigs being positively diagnosed with sub-clinical ASF infection [19].

Subsequent outbreaks have continued to stifle the pig industry in terms of sustainable commercial pig farming in Uganda. As accurate prediction of future ASF outbreak cycles and patterns in Uganda hinges on a clear understanding of past and current outbreak trends (Dr. Kiryabwire, DVO Mukono, personal communication), we sought to gain a clearer insight into the co-circulation of outbreak strains through a retrospective investigation.

The control and eradication of ASFV is difficult due to several factors including the absence of effective commercial vaccines, virus survival in the environment, viral resistance in infected tissues, contaminated material, and infectious animal products. In addition, ASF epidemiology is complex and its transmission involves the tick and wild pig reservoirs, as well as the domestic pigs and virus interactions [20]. To effectively institute quarantine and effective control measures, accurate and affordable diagnosis of ASF outbreaks is of paramount importance. We therefore designed this study to compare and contrast available yet sensitive and affordable diagnostic tools for ASFV detection, with emphasis on conventional PCR approaches.

## 2. Materials and Methods

### 2.1. Sample Collection and Acquisition

Using random field sampling, a total of 78 tissue samples from 59 animals were collected from seven districts (Pallisa, Lira, Abim, Nebbi, Kabarole, Kibaale, and Mukono) in Uganda in December 2012, during the outbreaks of ASF. All samples were obtained from the farm slaughter slabs, abattoirs, or from post-mortem procedures. Tissues were collected in labelled 10 mL Falcon tubes or vacutainers and stored at 4 °C until further use. For serology, 28 pigs were randomly sampled from each district. Four millilitres (4 mL) of blood was drawn from the jugular vein of pigs following manual restraint, and collected into red-capped (non-heparinized) vacutainer tubes (Becton Dickinson, Franklin Lakes, NJ, USA). These were transported to the laboratory and centrifuged at 2000 rpm for 15 min. Sera were decanted in duplicate and stored in well-labelled cryogenic vials at the Animal Virology Unit in NaLIRRI. All sample collections were done by veterinarians or animal health officers following ethical standards as approved. A total of 196 sera were collected from the seven districts. For the molecular assays, and in consideration of the then-ongoing widespread ASF outbreaks at the time of sampling, it was assumed that sampling would be from a large population, hence we purposively sampled farms and randomly sampled pigs in farms; we targeted a minimum of one and maximum of 13 pigs or carcasses per district, assuming 20% sensitivity of the virus detection test, for districts with pig populations ranging from 450 to over 100,000 pigs [16,21]. All samples were transported to the Infectious Animal Disease Laboratory (IADL) in NaLIRRI, and tissue samples were later purposively selected to ensure representation of all districts (n = 78). Tissue and serum samples were packaged and shipped to the OIE Reference Laboratory for ASF, the Transboundary Animal Disease Programme, Agricultural Research Council- Onderstepoort Veterinary Institute, South Africa, under the UN standards for transport of infectious material in packaging the materials ((UN2900; https://iris.who.int/bitstream/handle/10665/339825/9789240019720-eng.pdf?sequence=1), accessed 23 March 2013).

### 2.2. Antibody Detection ELISA for Recognition of ASF Antibodies

Serological analyses were performed at the Onderstepoort Veterinary Institute, South Africa, using a blocking enzymatic immunoassay (Blocking ELISA) kit. The antigen coated to the plate in the immunoassay kit consisted of purified virus protein 73 (VP73), which is the major structural protein from the ASFV and the most antigenic one (Ingezim PPA Compac, Ingenasa Spain), according to manufacturer’s instructions. Validation of the test for each plate was considered valid when the optical density (OD) of the negative control (NC) was at least four times higher than the OD of the positive control (PC). Two OD readings were obtained, and the mean OD was recorded for each sample. The positive and negative cut-off points were calculated using the following formulae, respectively: Positive cut-off = NC − [(NC − PC) × 0.5], Negative cut-off = NC − [(NC − PC) × 0.4], where NC corresponds to the OD of the negative control serum and PC corresponds to the OD of the positive control serum. Serum samples with an OD lower than the PC were considered positive to ASFV antibodies, and serum samples with an OD higher than the NC were considered negative to ASFV antibodies. Serum samples with OD values between both cut-offs were considered doubtful, and this study did not have any in that category.

### 2.3. OIE(WOAH)-Recommended Diagnostic PCR for ASF

#### 2.3.1. Extraction and Genomic Amplification of the Viral DNA

DNA extraction was conducted on a subset of 59 samples (40 single-tissue (liver, lung, kidney, mesenteric lymph node, heart, and spleen) and 19 pooled-tissue). This was performed using the prescribed protocol for nucleic acid extraction from mammalian tissue using the Roche High Pure PCR Template Preparation Kit (version 16.0). The 59 DNA templates were screened by conventional PCR using OIE prescribed forward primer ASF-1 (ATGGATACCGAGGGAATAGC) and reverse primer ASF-2 (CTTACCGATGAAAATGATAC) that target a 278 bp fragment of the ASF *p72* gene [22]. Products were run on a 1.5% agarose gel (Roche, Midrand, South Africa) stained with ethidium bromide and visualised under UV.

In addition, four gene regions of the ASF genome were targeted for multi-locus sequence analysis [23]. All reactions were performed using a touchdown PCR thermal cycling approach in combination with Biotools *Taq* polymerase (1U/reaction). The four gene regions were the *p72* gene, *p54* gene, the central variable region (CVR) of the *9RL* ORF and *TK* gene. Each primer set was assigned a single letter code, viz.: [P] *p72*-U + *p72*-D [4]—targeting a 478 bp region of the C-terminal end of *p72*, [A] PPA89 + PPA722 [24]—targeting a 257 bp region of *p54*, [C] CVR-FLF + CVR-FLR [24,25]—targeting the CVR of the *9RL* ORF [24], and [T] *TK*-1 + *TK*-Rev [25]—targeting the thymidine kinase gene. Reactions were performed in a final volume of 50 µL containing each of the primers at a final concentration of 0.4 µM, in the presence of 1U of Biotools *Taq* polymerase.

A touchdown PCR was performed on a gradient thermal cycler, with the following annealing temperatures and number of cycles: [A] 57 °C × 2; 56 °C × 3; 55 °C × 35, [C] 54 °C × 2; 53 °C × 3; 52 °C × 35, [P] 52 °C × 2; 51°C × 3; 50°C × 35 and [T] 49 °C × 2; 48 °C × 3; 47 °C × 35. All annealing steps were preceded by denaturation at 96 °C for 12 s, and followed by an extension/elongation step at 70 °C for 1 min.

#### 2.3.2. PCR Purification and Sanger Sequencing

All PCR products were run on a 1.5% agarose gel and sized against the 1 kbp Generuler (Thermo Fisher Scientific, Johannesburg, South Africa). Products of the expected size were purified directly from the tube using Roche High Pure PCR Purification Kit (Roche Diagnostics GmbH, Penzberg, Germany). All *p72*, *p54*, CVR, and *TK* (PACT) amplicons were sequenced with each of the corresponding PCR primers in separate reactions at an annealing temperature of 50 °C, 54 °C, 52 °C, and 48 °C, respectively, using BigDye version 2.0. Unincorporated nucleotides and primers were removed by ethanol precipitation with sodium acetate and submitted to the core Sanger sequencing at the University of Pretoria, South Africa.

### 2.4. Sequencing and Phylogeny

Sequences were edited, aligned, and phylogenetic analyses carried out using MEGA 7 [26] and Geneious version 10.0 [27]. Each individual gene dataset was complemented with homologous reference sequences identified through nucleotide BLAST searches against the Genbank database (https://blast.ncbi.nlm.nih.gov/), accessed 15 December 2023. Summary statistics and the best-fit model of sequence evolution were generated for each of the datasets; these guided phylogenetic inference for each gene region.

## 3. Results

### 3.1. Subsection

From the seven districts, a total of 193 sera were tested by blocking enzymatic immunoassay (ELISA); only six serum samples from the Abim district were positive. All sera from other locations were negative (Table 1).

### 3.2. Molecular Detection of African Swine Fever Virus

**Figure 1:**
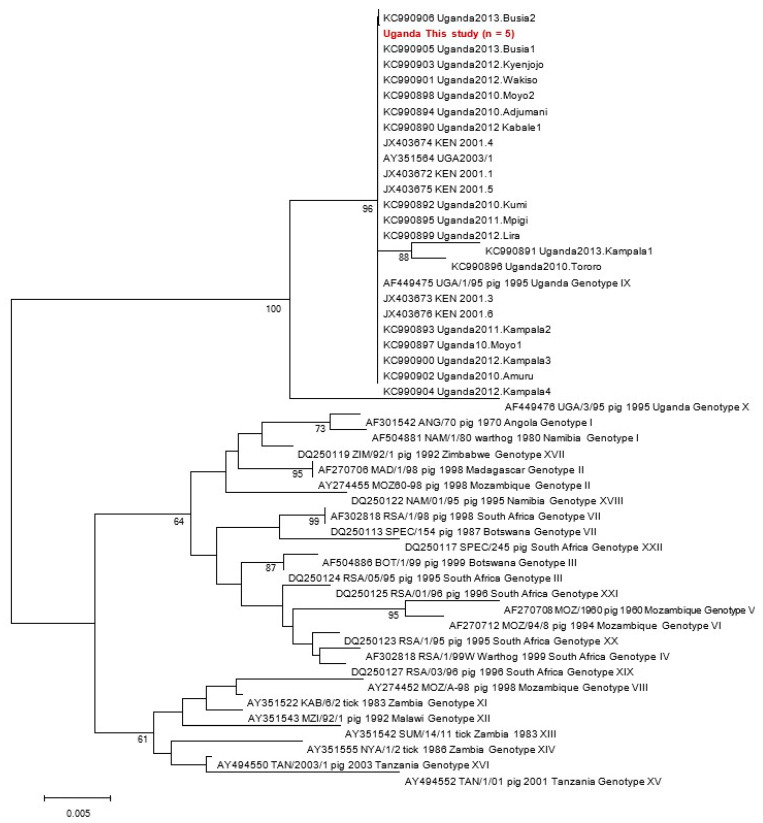
Neighbour-joining *p72* gene tree confirming the genotype IX assignment of ASF viruses characterised in this study (denoted in red). All five samples were identical across the C-terminal region that was sequenced. Bootstrap support values ≥ 60% are indicated next to the relevant nodes.

**Figure 2:**
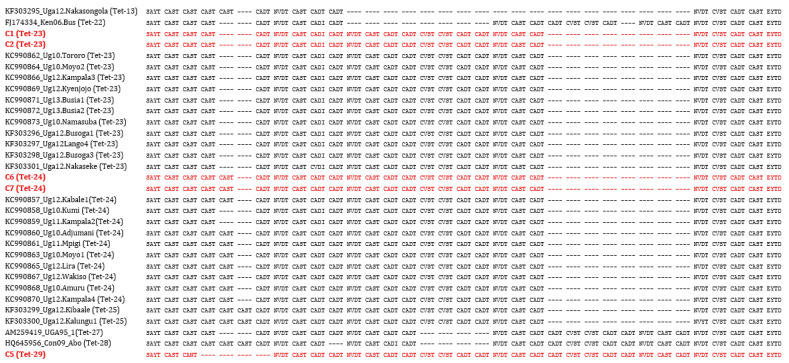
Tetramer alignment of the CVR of the *9RL* ORF (C1, C2, C5, C6, C7, indicated in red bold are derived from this study).

**Figure 3:**
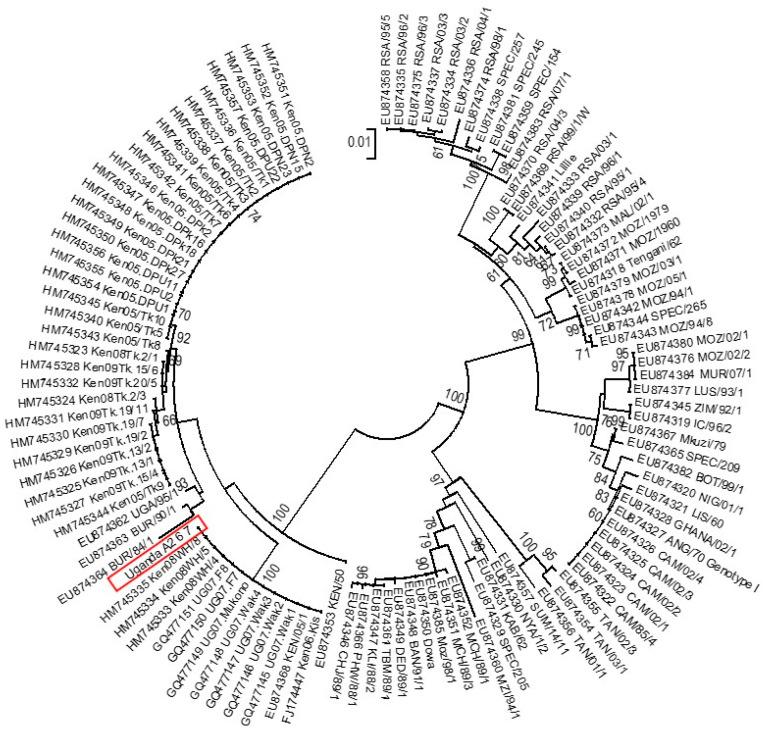
Neighbour-joining *p54* tree depicting the phylogenetic placement of the A-amplicon genotype IX sequences, represented by A2 (red box). Nodal support values ≥60 are indicated next to the relevant nodes.

**Figure 4:**
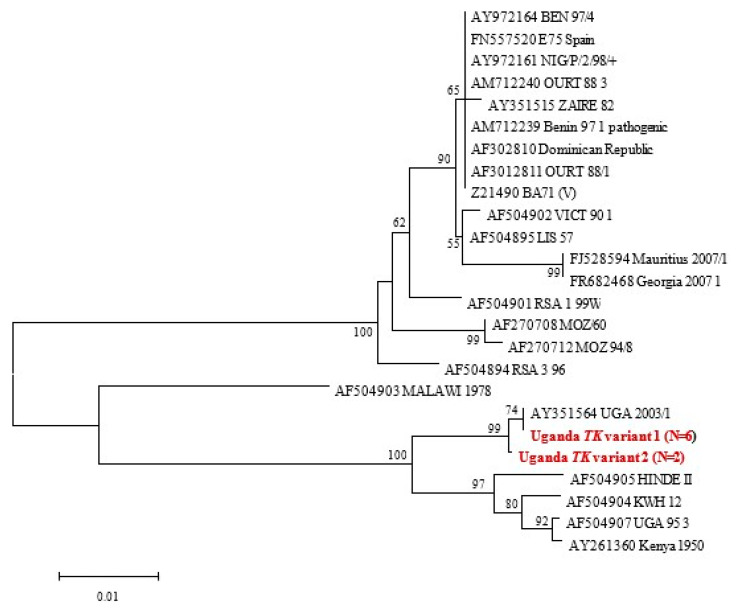
Neighbour-joining *TK* gene tree. Viruses indicated in red and bold correspond to samples characterised in this study. Nodal support values >70% from 1000 bootstrap replications are indicated next to the relevant nodes. Uganda TK variant 1 aligns with the Uganda 2003/1 virus, while the Uganda TK variant 2 is different from the first TK variant.

Briefly, 59 tissue samples were evaluated using five conventional PCR assays, including the WOAH (OIE)-recommended diagnostic assay that targets the central region of the *p72* gene. Sequencing of the C-terminal *p72* and complete *p54* gene regions confirmed that all viruses belonged to genotype IX, and the majority of strains were identical to one of the 1995 Ugandan ASFV outbreak strains [Table 2, Figure 1, Figure 2 and Figure 3; Supplementary Figures S1–S3]. Alignment of tetramers of the CVR within the *9RL* ORF recovered three CVR variants for the five positive amplicons; two had 23 tetramers, two had 24 tetramers and one (C5) had 29 tetramers (Figure 2). The initial screening with the *p72* gene revealed the presence of ASF viruses in four districts, namely Pallisa, Nebbi, Kibaale, and Mukono. At this stage, no viruses were detected in Lira, Abim, and Kabarole. Sequencing confirmed that all viruses belonged to genotype IX (Figure 1, Figure 3 and Figure 4), as detailed below:

#### 3.2.1. Conventional PCR Using *p72* Gene for ASF Diagnosis

The diagnostic PCR that targets a 278 bp region of *p72* identified seven positive samples from the 59 DNA extracts evaluated (11.86% PCR positivity; Supplementary Figure S1; Table 2).

#### 3.2.2. Multilocus Typing of the Ten Positive DNA Extracts Using a Four Gene Region (PACT) Approach

Using touchdown PCR, we attempted amplification of four gene regions in duplicate for all samples identified as ASF-positive by diagnostic PCR. As the TK primer assay was the only typing assay that amplified all seven positive samples, and as amplification success varied by assay, it became essential to re-evaluate all 59 DNA extracts using the TK-1 and TK-Rev primers. After the re-evaluation analysis using the TK-1 and TK-Rev primers, three new positive amplicons (2a, 15, and 17) were identified that were originally negative by all the other methods, resulting in 10 positives in total (Table 2, Supplementary Figures S2–S4). The number of positives remained unchanged after re-evaluation of all 59 extracts with the P, A, and C assays.

### 3.3. Molecular Diagnosis of African Swine Fever Virus Phylogenetic Analyses

All positive PCR products were purified and submitted for Sanger sequencing. The resulting chromatograms were viewed, edited, and aligned in MEGA8, and each dataset was complemented with published sequences sourced from the Genbank database (www.nlm.nih.gov), accessed on 15 December 2023. Individual gene phylogenies were inferred using the best fit model of sequence evolution identified under the Bayesian Information Criterion (BIC) in MEGA8 [26].

#### 3.3.1. *p72* Gene Phylogeny

All five positive samples were sequenced, and BLAST nucleotide searches against the Genbank database identified a number of identical and near-identical genotype IX African swine fever viruses from Uganda and Kenya (Figure 1).

#### 3.3.2. CVR- *9RL* ORF Tetramer Alignment of the Positive 5 Amplicons

The five ASF-positive sequences identified by CVR- 9RL ORF amplification and sequencing were translated. Each of the three variants were used in BLAST nucleotide searches against the Genbank database (https://blast.ncbi.nlm.nih.gov/Blast.cgi), accessed on 15 December 2023, to identify related, homologous sequences. The predicted amino acid sequences were visually aligned by tetrameric repeat, as captured in Figure 2. The Tet-23 and Tet-24 variants have previously been reported from Uganda, but Tet-29 has not.

#### 3.3.3. *p54* Gene Phylogeny

Three of the four amplicons confirm genotype IX virus assignment. The viruses characterised in this study are identical to each other and denoted by the Uganda A2 representative sequence (in red box; Figure 3), which clusters within a well-supported clade (100% bootstrap support) together with viruses from Kenya and Uganda.

#### 3.3.4. *TK* Gene Phylogeny

Following nucleotide sequence alignment of the eight viruses characterized in this study (Table 2), two *TK* gene variants (differing from each other at a single nucleotide site) were detected, and their relationship to available homologous data was inferred as shown in Figure 4. *TK* variant 2 (TK-V2) was detected at the Kibaale sampling site (Samples T3 and T5).

#### 3.3.5. Combined PACT Results

The combined sequencing results identified three distinct strains (a-c) based on their P-A-C-T typing results, viz. (a) IX, IX, Tet-23, TK1-V1; (b) IX, IX, Tet-24, TK-V1 and (c) IX, IX, Tet-29, TK1-2.

## 4. Discussion

### 4.1. Determination of ASF Outbreaks Using Serology

In Uganda, African swine fever is endemic, and has continued to stifle the pig industry in terms of sustainable commercial pig farming. As no readily available and effective ASF vaccines exist, outbreaks can only be controlled through prompt detection and quarantine [15,18]. To effectively establish quarantine measures for effective control, accurate, affordable, and rapid diagnosis of ASF outbreaks is vital. Following several reports citing pig deaths in Uganda, we undertook this work to directly associate pig deaths to ASF. We further aimed to compare the available serological and molecular diagnostic options which several laboratories in Uganda could co-opt or introduce affordably as methods for ASFV detection. The cheaper and less-invasive method for confirmation of ASF is the ELISA serology. Our study used and compared five tests to confirm the presence of ASF in Uganda. Sera originated from seven districts in Uganda: Pallisa, Nebbi, Kibaale, Mukono, Kabarole, Abim, and Lira. The ELISA only detected antibodies from pigs in the Abim district, leaving out the other six districts that were confirmed positive using molecular tests and the TK assay, in particular, in this study. It should be noted that ASF outbreaks happened in the Abim district far earlier than in other districts, prior to our sampling. Neutralizing antibodies may have had sufficient time to develop compared to the other districts, where outbreaks were ongoing during the period of sampling. Numerous other studies have confirmed similar observations wherein sera collected from clinically sick pigs and tested using the prescribed OIE serological tests were negative [22,26].

Perez-Filguera et al. [20], using both the recombinant and conventional ELISAs on the p30r protein, showed variable rates of sensitivity and specificity with African samples originating from different geographies. Most likely, the differential rates in the work above may be associated with antigenic variations among isolates; producing antigenically-specific versions of p30r targeting ASFV serotypes more distant to genotype I and incorporating them in the antigen preparation may become expedient. Gallardo et al. [20,24] attributed the unexpectedly low seropositivity in East African originated pig sera, especially when the OIE-prescribed methods are used, to the immunogenetics of the indigenous pig populations and not the polymorphisms in immunodominant viral antigens. Since ASF is a rapidly fatal disease, infected domestic pigs usually die before the development of antibodies, or are culled or sold off to the market, a practice that is rife amongst smallholder pig farmers in Uganda. Muwonge et al. [28] also noticed that farmers in Mubende slaughtered a large number of untested pigs during an outbreak to minimise the losses from pig deaths. This would explain the lower antibody titres detected, implying serology may not be a good indicator of ASF status during an active outbreak. Typically, pigs that survive natural infection usually develop antibodies against ASFV from 7–10 days post-infection, and these antibodies persist for long periods of time.

Limited serological assays conducted for ASF in other locations confirmed a prevalence of 2.1% and 0.2% in Uganda [15,23], and 9% in Nigeria [29]. The genetic analysis of samples taken from the same sites (and animals) were positive even where all sera were negative. In this study, using serology alone as a definitive diagnostic tool in an ongoing outbreak of ASF may miss approximately 86% of potentially positive samples (calculated from 6 out of 7 districts considered negative by ELISA, yet positive by PCR). In common with other authors, we conclude that ASF antibody detection may not be a good indicator of the field situation during an ongoing outbreak or in the immediate post-outbreak period. Recent anecdotal evidence from the field suggests that outcomes of serological testing may be country- and context-specific for ASFV [20,22]. In Uganda, the twin situation of ongoing outbreaks and endemicity of the disease makes it difficult to distinguish between endemic ‘silent’ spread and new infections. A similar survey conducted in Senegal showed that the presence of ASF antibodies can only be an indication of a previous field-level encounter with ASF, and not an indicator of current infection [22,28,29,30]. Our results concur with the conclusion above, although they do not inform the specific period of occurrence of the past outbreaks. Hutchings and Ferris [31] have earlier suggested that ELISA serology alone should not be used as a diagnostic test because it may not be able to detect low concentrations of antibodies, especially from poor-quality diagnostic material. This becomes beneficial if clinical samples are considered with regards to virus concentrations in tissues, and individual pigs’ viral loads. A country’s surveillance team’s dependence on serology only, as a basis for definitive diagnosis, may inadvertently support the spread of ASF.

### 4.2. Determination of ASF Outbreaks Using Molecular Tools

The TK gene performed better by amplifying 10 targets in total, three of which were new detections missed by an earlier round of screening with the diagnostic PCR that targets the *p72* gene (Figure 4, Table 2). Reasons for this include a possible change in the genome diversity and strain variability of the ASFV or greater sensitivity offered by the TK gene. Two new sequence variants (Figure 4) were recovered using the TK gene phylogeny, within which a single nucleotide mutation was detected, which introduces a premature stop codon resulting in a truncated TK protein 185 aa in length instead of 196 aa. One of the new TK variants from our study, the Uganda Tk variant 1, has 74% similarity with a previously published AY35564 UGA 2003/1 virus that caused an outbreak in 2003 in Uganda. It would be interesting to find out what these nucleotide differences translate into in terms of virus pathogenicity and detection. From the outbreak distribution map in Uganda, previously published by Kabuuka et al. [32,33], the two Uganda TK variants seem to originate in the mid-northern belt of the country and in the Totoro district of Uganda [34], with no clear explanation yet for this observation [32].

In a phylogenetic study of ASF outbreaks in Uganda from 2001–2012, Atuhaire et al. [18] detected 21 viruses out of the 30 outbreaks, saying that the nine undetected could have been due to another aetiology, since PCR is highly sensitive. We found that the two *p72* gene assays used in this study were not able to detect three variants from the Abim, Lira, and Nebbi districts (Table 2). The *TK* gene readily detected these viruses and also detected two more, which were positive for the *p72* gene and negative with the *p54* gene primers. We hypothesize that the viral load in the field samples could be low in concentration (although we did not carry out DNA template ranging), hence the low levels of amplification, or that viruses with different virulence could be co-circulating. Since the *TK* gene is associated with virulence, we think that it is useful in outbreak situations for detecting variant strains. The fact that ASF may be under-diagnosed creates a challenge for control, since such viruses will be transmitted inadvertently to areas where no control is being implemented. More studies are needed to confirm the reliability of the TK assay as an additional molecular diagnostic marker for African swine fever detection, particularly in low-resource countries where real-time PCR capabilities are limited. Acknowledged limitations of the study include the number of districts covered and the total number of samples processed may be insufficient to draw robust conclusions, especially given the geographic diversity of Uganda and virus circulation [35]. Future studies should consider the expansion of the study areas to include more districts and consider a larger sample size to draw inference for generalizability. In addition, it should be noted that this work is exploratory. Replication studies in other locations in Uganda and elsewhere are crucial to validate the findings in this work.

## 5. Conclusions

Findings from our molecular experiments confirm the endemicity of ASF in Uganda and show the superiority of molecular assays over serological ones. A new TK gene PCR detected more outbreaks than the formerly prescribed *p72* diagnostic PCR, and we identified two TK variants, namely Uganda TK variant 1 and Uganda *TK* variant 2, both belonging to genotype IX viruses and associated with CVR tetramer variants C1 (Tet-23), C2 (Tet-23), C6 (Tet-24), C7 (Tet-24), and C5 (Tet-29), respectively. Repeatability tests are needed to confirm TK gene performance as an additional molecular diagnostic marker for African swine fever detection in Uganda or East Africa. Our results further confirm variation in the endemic circulating strains of ASF in Uganda, with varying implications for future diagnosis and vaccine development efforts.

## Figures and Tables

**Table 1:** Serology results of samples collected from the seven districts of Uganda.

District	Samples Positive (%)	Minimum Optical Density	Maximum Optical Density	Samples Negative (%)	Minimum Optical Density	Maximum Optical Density
Pallisa	0 (0)	-	-	28 (100)	1.876	2.509
Lira	0 (0)	-	-	28 (100)	1.901	2.349
Abim	6 (24)	0.058	0.739	19 (76)	1.749	2.254
Nebbi	0 (0)	-	-	28 (100)	1.844	2.489
Kabarole	0 (0)	-	-	28 (100)	1.815	2.519
Kibaale	0 (0)	-	-	28 (100)	1.9	2.736
Mukono	0 (0)	-	-	28 (100)	1.905	2.55
Total	6 (3.1)			187 (96.9)		

Of the 196 sera collected, only 193 were processed and run. Three samples were excluded as they were unsuitable for ELISA serology due to haemolysis.

**Table 2:** ASF Diagnostic test matrix of 10 matching samples from pigs highlighting performance of the WOAH (OIE) Diagnostic PCR and the four evaluated gene regions used for virus typing.

District of Origin	Laboratory Sample Name	Number Assigned for Multilocus Typing	Tissue Type	WOAH (OIE) Diagnostic PCR	Four Primer Sets			
					[A] PPA89 + PPA722	[C] CVR-FLF + CVR-FLR (Tetramer Number)	[P] P72-U + p72-D	[T] * TK1 + TK-rev
Pallisa	10	1	Kidney	+	A1	C1 (Tet-23)	P1	T1
Nebbi	36	2	Spleen	+	A2	C2 (Tet-23)	P2	T2
Kibaale	46	3	Liver	+	-	-	-	T3 *
Kibaale	48	4	Mesenteric lymph node	+	-	-	-	T4 (NS)
Kibaale	52	5	Spleen	+	-	C5 (Tet-29)	P5	T5 *
Mukono	58	6	Liver	+	A6	C6 (Tet-24)	P6	T6
Kibaale	59	7	Liver	+	A7	C7 (Tet-24)	P7	T7
Nebbi	2a	2a	Spleen	-	-	-	-	T2A (NS)
Abim	15	15	Mesenteric lymph node	-	-	-	-	T15
Lira	17	17	Kidney	-	-	-	-	T17
		PCR-positivity		11.86%	6.78%	8.47%	8.47%	16.95%
		Relative sensitivity (compared to TK)		70%	40%	50%	50%	-
Degree of agreement with diagnostic primers (%) based on Kappa statistics					70	80	80	100
Kappa statistics					0.44 (moderate)	0.60 (moderate)	0.60 (moderate)	0.00 (slight)

Column 5 summarises the positive amplicons originally detected by conventional PCR with the WOAH (formerly OIE) diagnostic primers (ASF-1 and ASF-2), which were subsequently compared with the four sets of primers targeting the *p72* (P), *p54* (A), CVR (C), and thymidine kinase (T) genes, the so-called “PACT” multi-locus sequence analysis scheme (rows 8–10 provide details of three additional positive amplicons detected using the TK primers, which were negative by WOAH diagnostic primer screening). * Indicates TK variant 2. NS: Not sequenced.

## Data Availability

All supporting data used in this research are freely available as Supplementary Materials or at the UPeTD (https://repository.up.ac.za/handle/2263/31741, accessed on 15 December 2023). All sequence data are available in the manuscript with their Accession numbers.

## Supplementary Materials

The following supporting information can be downloaded at: https://www.mdpi.com/article/10.3390/ani14010071/s1, Figures S1–S4: Figure S1: Agarose gel electrophoresis of p72-PCR products amplified with P1 and P2 OIE p72 screening primers (Lane m: 100 bp ladder; lane N: Negative control; lane P: Positive control; lanes 10, 36, 46, 48, 52, 58 and 59; Positive amplicons (n = 7)); Figure S2: Agarose gel electrophoresis of p54 [A], CVR-ORF [C], p72 [P] and Tk [T] gene cycle sequenced products using modified reaction conditions and demonstrating improved TK gene amplification (Lanes L = 100 bp ladder; N = Negative control; A1, P1, P6, T1, T2, T3, T4, T6 and T7 are positive amplicons from purified DNA products. The figure showed that agarose gel bands for positive cycle sequenced products are evident in lanes in A1, P1, P6, T1, T2, T3, T4, T6 and T7. Figure 3 shows the second PCR results in lanes A2, A6, A7, C1, C2, C5, C6, C7, P1, P2, P5, P6, P7, T2, T5, T6 and T7); Figure S3: Agarose gel electrophoresis of p54 [A], CVR-ORF [C], p72 [P] and Tk [T] gene products (Lanes L = 100 bp ladder; N = Negative control; A2, A6, A7, C1, C2, C5, C6, C7, P1, P2, P5, P6, P7, T2,T5, T6 and T7 are positive amplicons from purified DNA products); Figure S4: Agarose gel electrophoresis of TK gene-PCR products amplified with TK-1 + TK-Rev primers (Lane L = 100 bp ladder; N = Negative control; P1= Positive control; 2, 15 and 17 are new Tk gene positive amplicons).

## Acronyms

ASF = African swine fever; ASFV = African swine fever virus; CVR = central variable region; DNA = Deoxyribonucleic acid; DVO = District Veterinary Officer; ELISA = Enzyme-linked immunosorbent assay; FABI = Forestry and Agricultural Biotechnology Institute; IADL = Infectious Animal Disease Laboratory; Mab = Monoclonal antibodies; NaLIRRI = National Livestock Resources Research Institute; NC = Negative control; ORL = Open reading frame; OD = Optical density; PC = Positive control; PPA = La peste porcina Africana; PCR = polymerase chain reaction; TK = Thymidine kinase; VP 73 = Virus protein; WOAH = World Organisation for Animal Health.
