# Supplementary material for: Retrospective Multi-Locus Sequence Analysis of African Swine Fever Viruses by “PACT” Confirms Co-Circulation of Multiple Outbreak Strains in Uganda
Source: Animals (Basel). 2023 Dec 24;14(1):71. doi: 10.3390/ani14010071 (PMC10777903; doi:10.3390/ani14010071)
Supplement: Supplementary file 1 [file animals-14-00071-s001.zip › animals-2776206-supplementary.pdf]

Supplementary materials

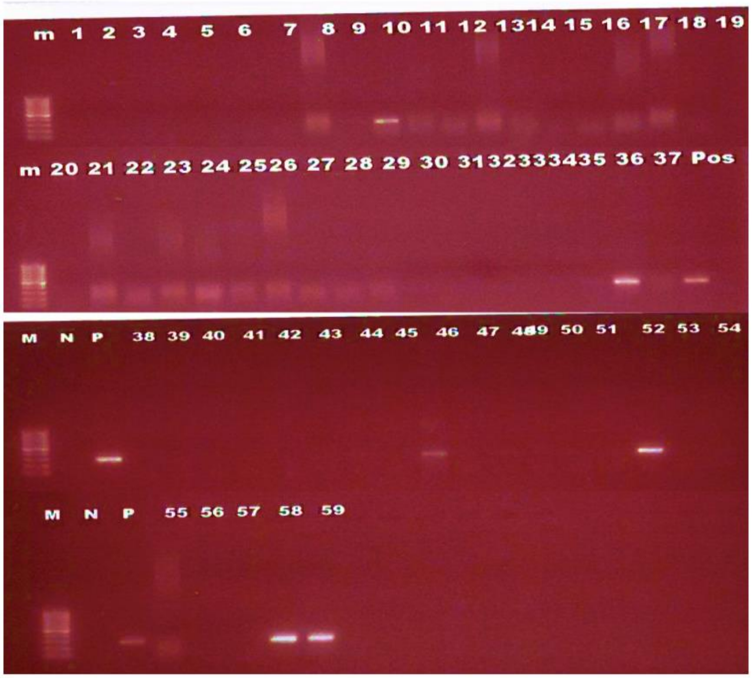

Supplementary Figure S1: Agarose gel electrophoresis of *p72*-PCR products amplified with P1 and P2 OIE *p72* screening primers.

Lane m: 100 bp ladder; lane N: Negative control; lane P: Positive control; lanes 10, 36, 46, 48, 52, 58 and 59; Positive amplicons (n = 7).

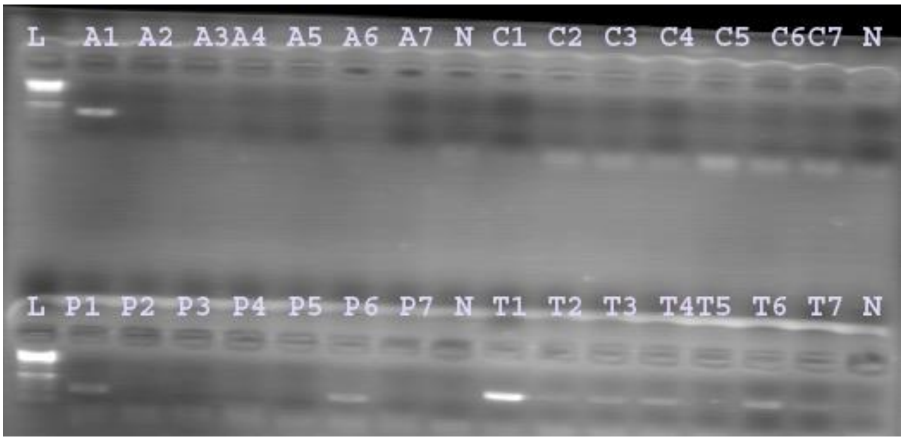

Supplementary Figure S2. Agarose gel electrophoresis of *p54* [A], CVR-*ORF* [C], *p72* [P] and *Tk* [T] gene cycle sequenced products using modified reaction conditions and demonstrating improved *TK* gene amplification. Lanes L = 100 bp ladder; N = Negative control; A1, P1, P6, T1, T2, T3, T4, T6 and T7 are positive amplicons from purified DNA products.

The figure 2 above shows that agarose gel bands for positive cycle sequenced products are evident in lanes in A1, P1, P6, T1, T2, T3, T4, T6 and T7. Figure 3 shows the second PCR results in lanes A2, A6, A7, C1, C2, C5, C6, C7, P1, P2, P5, P6, P7, T2, T5, T6 and T7

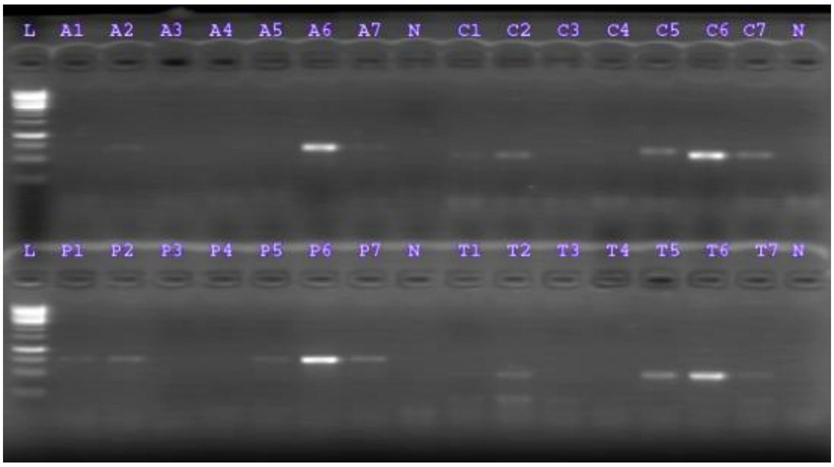

Supplementary Figure S3. Agarose gel electrophoresis of *p54* [A], CVR-*ORF* [C], *p72* [P] and *Tk* [T] gene products. Lanes L = 100 bp ladder; N = Negative control; A2, A6, A7, C1, C2, C5, C6, C7, P1, P2, P5, P6, P7, T2,T5, T6 and T7 are positive amplicons from purified DNA products.

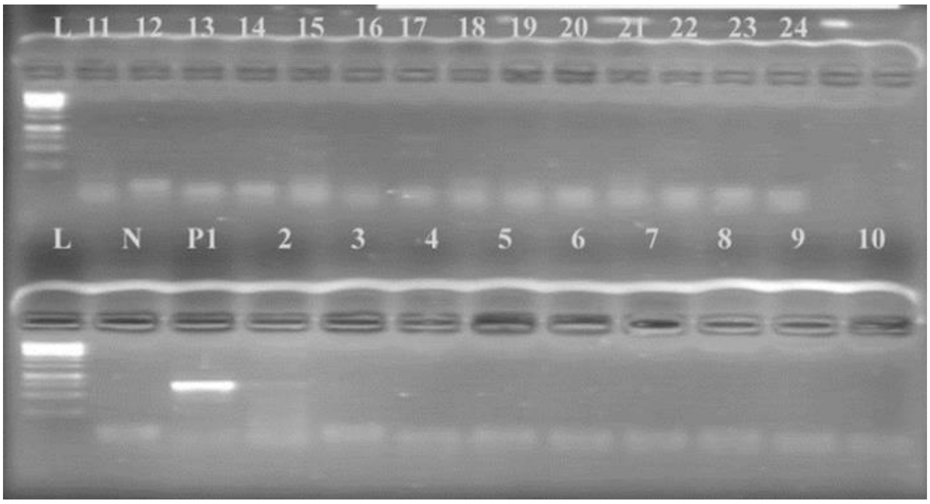

Supplementary Figure S4. Agarose gel electrophoresis of *TK* gene-PCR products amplified with *TK*-1 + *TK*-Rev primers. Lane L = 100 bp ladder; N = Negative control; P1= Positive control; 2, 15 and 17 are new Tk gene positive amplicons.
